# Radiographic Evaluation in Alveolar Preservation Using Platelet-Rich Fibrin: A Randomized Controlled Trial

**DOI:** 10.3390/dj13060231

**Published:** 2025-05-23

**Authors:** Magdalena Molina-Barahona, Jordano Castillo, Esteban Freire-Meza, Ana Cristina Vásquez-Palacios, Denia Morales-Navarro, Renata Avecillas-Rodas

**Affiliations:** 1 Maxillofacial Radiology Department, Faculty of Dentistry, Universidad Católica de Cuenca, Cuenca 010107, Ecuador; 2 Periodontics and Surgical Implantology, Universidad Católica de Cuenca, Cuenca 010107, Ecuador; jordano.castillo@est.ucacue.edu.ec (J.C.); esteban.freire.55@est.ucacue.edu.ec (E.F.-M.); avasquezp@ucacue.edu.ec (A.C.V.-P.); 3 Faculty of Dentistry, Universidad Católica de Cuenca, Cuenca 010107, Ecuador; renata.avecillas@est.ucacue.edu.ec; 4 Maxillofacial Surgery Department, Universidad de Ciencias Medicas de la Habana, Habana 104000, Cuba; denia.morales@infomed.sld.cu

**Keywords:** platelet-rich fibrin, bone regeneration, socket preservation, CBCT, alveolar ridge

## Abstract

**Background**: Tooth extractions commonly result in dimensional changes of the alveolar ridge. Platelet-rich fibrin (PRF) has emerged as a promising autologous biomaterial for alveolar preservation. This randomized controlled trial aimed to evaluate, through cone beam computed tomography (CBCT), the effect of PRF in maintaining alveolar dimensions post-extraction. **Methods**: A single-blind, randomized controlled clinical trial was conducted in 10 systemically healthy patients requiring premolar extractions for orthodontic reasons. A total of 36 alveoli were analyzed: 19 with PRF (experimental group) and 17 without PRF (control group). CBCT scans were performed at baseline, 30 days, and 120 days post-extraction to measure alveolar height, vestibulo-palatal/lingual depth at 1, 3, and 5 mm, and bone tissue density using Hounsfield Units (HU). **Results**: Baseline cephalocaudal alveolar height was similar in both groups (~10.5 mm, *p* = 0.399). At 30 days, height preservation was significantly greater in the PRF group (10.61 mm vs. 8.82 mm, *p* < 0.001). At 120 days, the PRF group maintained greater height (10.30 mm vs. 9.31 mm), although this difference was not statistically significant (*p* = 0.059). No significant differences were observed in alveolar depth at 1, 3, or 5 mm (*p* > 0.05). The PRF group showed a trend toward better preservation and higher mean bone density values (190–282 HU), although no formal statistical comparison of HU values was performed. Repeated measures ANOVA revealed a significant interaction effect of time and group on alveolar height (*p* = 0.010, η^2^ = 0.046) and at 1 mm depth (*p* = 0.035, η^2^ = 0.020). **Conclusions**: PRF significantly improved short-term alveolar height preservation. Trends toward better depth preservation and higher bone density values were observed in the PRF group, although these findings were not statistically significant. PRF appears to be a safe biomaterial with potential to support alveolar ridge maintenance post-extraction.

## 1. Introduction

Tooth extraction inevitably leads to dimensional changes in the alveolar ridge due to the remodeling of hard and soft tissues. Within the first 3 to 6 months, horizontal ridge width may decrease by 2.6–4.6 mm and vertical height by 0.4–3.9 mm, with up to 60% of the original volume lost in the first year [1,2]. These changes can compromise future implant placement, esthetic outcomes, or orthodontic planning.

While conventional socket preservation techniques using bone grafts or membranes are effective, they often involve additional cost, foreign materials, and extended healing times [3]. Platelet-rich fibrin (PRF), a second-generation autologous platelet concentrate, has emerged as a promising alternative due to its ability to promote soft and hard tissue regeneration without additives or anticoagulants [4,5]. PRF provides a fibrin scaffold rich in leukocytes and platelets, supporting angiogenesis and osteogenesis through the gradual release of growth factors such as PDGF and TGF-β [6,7,8].

Several studies have suggested that PRF may reduce vertical ridge resorption and improve bone healing compared to natural healing [9,10]. However, the evidence regarding its impact on horizontal dimensional changes and bone tissue quality remains limited and somewhat inconsistent [11]. Moreover, most clinical trials have focused on implant-related extractions in older patients, whereas few studies have evaluated PRF in young adults undergoing premolar extractions for orthodontic purposes—a scenario that represents potentially optimal healing conditions.

In this context, the present study aimed to assess the potential clinical utility of PRF for alveolar ridge preservation, using high-resolution cone beam computed tomography (CBCT). This imaging modality allows for precise three-dimensional measurements of ridge dimensions and assessment of bone density through Hounsfield Units (HU), providing a non-invasive method to monitor regenerative outcomes [12].

Recent literature has explored the application of PRF in periodontal and oral surgical procedures. Silva et al. conducted a systematic review and meta-analysis supporting its use in periodontal surgery, highlighting its ability to enhance healing and reduce alveolar bone loss [13]. Pradeep et al. compared PRF and PRP in periodontal regeneration and found that PRF offered superior outcomes in both clinical and radiographic parameters [14]. Borie et al. provided an overview of PRF applications across dentistry, including oral surgery and implantology, noting its low cost, ease of preparation, and biocompatibility [15]. Furthermore, Del Corso et al. discussed its role in bone grafting and implant procedures, emphasizing its capacity to support bone regeneration through sustained release of growth factors and structural integration with host tissues [16].

Therefore, the rationale for this study is to evaluate the potential role of PRF in preserving alveolar ridge dimensions post-extraction, focusing primarily on vertical height preservation, with secondary assessment of horizontal width and descriptive analysis of bone density changes using CBCT.

### Objectives

Primary Objective: 

To evaluate the radiographic effectiveness of platelet-rich fibrin (PRF) in preserving alveolar ridge height following premolar extractions for orthodontic indications.

Secondary Objectives: 

To descriptively compare alveolar width changes (measured at 1 mm, 3 mm, and 5 mm from the crest) between PRF-treated and control sockets, acknowledging the lack of statistically significant differences.

To descriptively assess bone tissue density at the extraction sites based on Hounsfield Unit (HU) values obtained from CBCT imaging, without formal statistical comparison.

## 2. Materials and Methods

### 2.1. Trial Design

#### 2.1.1. Study Design

This study was designed as a single-blind, two-arm, parallel-group randomized controlled clinical trial with a 1:1 allocation ratio. The objective was to evaluate, through cone beam computed tomography (CBCT), the effectiveness of platelet-rich fibrin (PRF) in the preservation of alveolar ridge dimensions following premolar extractions indicated for orthodontic purposes. A total of 36 alveoli were included, allocated to an experimental group (*n* = 19) treated with PRF and a control group (*n* = 17) left to heal physiologically. The trial was conducted in accordance with the ethical principles of the Declaration of Helsinki and adhered to the Consolidated Standards of Reporting Trials (CONSORT) statement for reporting health research [17]. Prior to participation, all subjects were thoroughly informed about the study protocol, including potential risks and benefits, and provided written informed consent. Ethical approval was obtained from the Bioethics Committee of the Catholic University of Cuenca under protocol reference number CEISH-UCACUE-031. The study was prospectively registered on ClinicalTrials.gov (NCT: NCT06900036), available online: https://clinicaltrials.gov/study/NCT06900036 (accessed on 14 May 2025).

#### 2.1.2. Study Population

Inclusion Criteria:

Participants included systemically healthy individuals aged 17–38 years who required bilateral premolar extractions for orthodontic reasons. Additional inclusion criteria involved candidates for orthognathic surgery or third molar extraction with proximity to critical structures, justifying the use of CBCT.

Exclusion Criteria:

Participants with systemic diseases, immunosuppression, smoking, pregnancy or lactation, low platelet count (<200,000/mm^3^), or use of medications interfering with healing were excluded.

### 2.2. Sample Size and Randomization

Sample size was calculated using G*Power software (version 3.1.9.4, Heinrich-Heine-Universität Düsseldorf, Düsseldorf, Germany) based on repeated measures ANOVA with an effect size of 0.25, an alpha level of 0.05, and a power of 95%, resulting in a required total of 36 alveoli (17 control, 19 PRF). No interim analyses or stopping rules were applied.

Randomization was executed using Epidat software Statistical analyses were also performed using Epidat software, version 4.2 (Dirección Xeral de Saúde Pública, Xunta de Galicia, Santiago de Compostela, Spain), generating a simple random sequence without blocking or stratification. The allocation list was concealed until patient consent and surgical planning were completed. A biostatistician independent of the clinical team generated the random sequence. Participants were enrolled by the lead investigator, and allocation to intervention was performed by a separate clinician according to the concealed list.

This was a single-blind study where the radiographic evaluator, a maxillofacial imaging specialist, was blinded to group assignment. Due to the nature of the intervention, neither patients nor surgeons were blinded. However, the use of contralateral sites within the same patient reduced inter-individual variability and strengthened internal validity.

### 2.3. Surgical Procedure

Experimental Group (PRF):

Following venipuncture and centrifugation using a PRF Duo system (Nice BD Vacutainer^®^ Push Button Blood Collection Set, 23G x ¾″, 12″ tubing with Luer adapter (REF: 367342), Becton, Dickinson and Company, Franklin Lakes, NJ, USA), autologous platelet-rich fibrin (PRF) clots were obtained and applied to the extraction sockets. One PRF clot was used to fill the socket, while the second was flattened to serve as a guided tissue regeneration membrane. Both clots were stabilized with 3-0 catgut sutures.

Control Group:

Sockets were allowed to heal physiologically without the application of PRF or any additional biomaterial.

All surgical interventions were performed under local anesthesia using atraumatic extraction techniques. Test and control sites were treated simultaneously during the same surgical phase in each patient to minimize procedural variability.

PRF Preparation

PRF preparation was performed according to the protocol developed and patented by Choukroun et al. [4], utilizing the PRF Duo centrifuge system and its corresponding surgical kit. No biochemical manipulation of the blood was performed.

For each alveolus to be treated with PRF, 10 mL of venous blood was collected from the antecubital vein into two sterile, additive-free glass tubes (10 mL each). Immediately after collection, the tubes were centrifuged at 2700 rpm for 12 min. The tubes were then left undisturbed for 5 min. The PRF clot located between the red blood cell layer and the acellular plasma was extracted.

One PRF clot was used to fill the extraction socket, and the second was compressed using the surgical box (Zimmer Biomet Dental, Palm Beach Gardens, FA, USA) to create a membrane for guided tissue regeneration. The fibrin matrix was gently inserted into the socket, and the membrane was positioned over the alveolar crest. Both were stabilized with 3-0 catgut sutures using a simple interrupted technique to ensure stability during early healing.

### 2.4. Surgical Protocol

Both experimental and control sites were treated in the same surgical session.

After achieving local anesthesia, an intrasulcular incision was made using a No. 15 scalpel (Bard-Parker®, Aspen Surgical Products, 6945 Southbelt Dr SE, Caledonia, MI 49316, USA) blade. Maxillary or mandibular premolars were extracted atraumatically according to orthodontic indications.

At control sites, extraction sockets were allowed to heal physiologically without intervention. At experimental sites, PRF clots were partially distributed within the socket, and the PRF membrane was placed over the socket opening as a barrier to promote guided tissue regeneration. Simple interrupted sutures were placed to secure the materials and promote primary closure.

The only difference between the surgical procedures at the test and control sites was the use of PRF in the experimental group.

All surgical interventions and clinical measurements were performed by a multidisciplinary team comprising a maxillofacial surgeon, periodontists, and a maxillofacial imaging specialist, following the standardized study protocol.

#### 2.4.1. Postoperative Care

Sutures were removed 7 days after surgery. Clinical follow-up examinations were conducted at 3, 7, and 14 days postoperatively. No complications or adverse events were recorded during the follow-up period.

#### 2.4.2. Postoperative Radiographic Evaluation

Immediately following tooth extractions, baseline CBCT scans were performed using the Axeos CBCT system (Dentsply Sirona, Bensheim, Germany). imaging equipment. Follow-up CBCTs were acquired at 30 and 120 days post-extraction, maintaining identical technical and radiation protection parameters for all imaging sessions.

All scans were obtained using a field of view (FOV) of 8 × 8 cm and a voxel size of 0.2 mm. Exposure parameters were set at 16 s, 30.89 mAs, and 120 kVp.

Image reconstruction, visualization, and measurements were performed using Sidexis 4.0 software. All analyses were conducted by a board-certified maxillofacial radiologist who was blinded to group allocation.

### 2.5. Measurement Techniques

Alveolar Ridge Dimensions

**Figure 1:**
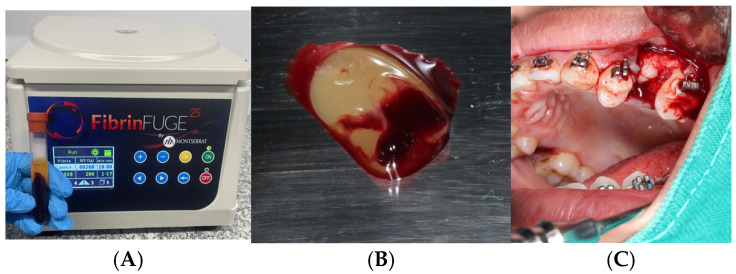
(**A**) Blood sample after centrifugation using the FibrinFUGE 25 (Montserrat^®^ Montevideo, Uruguay), showing the separation of the leukocyte- and platelet-rich fibrin (L-PRF) clot. The clot appears as the yellowish middle layer between the upper platelet-poor plasma and the lower red blood cell fraction. This stage precedes membrane preparation. (**B**). Atraumatic post-extraction socket of the maxillary left first premolar (tooth 2.4), following surgical removal for orthodontic purposes. The socket presents with intact cortical walls and minimal soft tissue trauma, providing a favorable site for PRF membrane placement. (**C**). Freshly obtained PRF clot prior to compression. The fibrin matrix appears well-formed with visible incorporation of red blood cells at the base. This intermediate step is essential before the formation of a membrane suitable for insertion into the extraction socket.

Following the method proposed by [18], measurements of alveolar dimensions were performed in both vertical and horizontal planes:

Depth Measurements (Vestibular–Palatal/Lingual):

The alveolar width was measured at three standardized depths: 1 mm, 3 mm, and 5 mm apical to the highest point of the vestibular cortical plate. Measurements were taken perpendicular to a vertical reference line drawn centrally within the alveolus from the apex to the alveolar crest, according to anatomical orientation (maxilla or mandible) (Figure 1).

**Figure 2:**
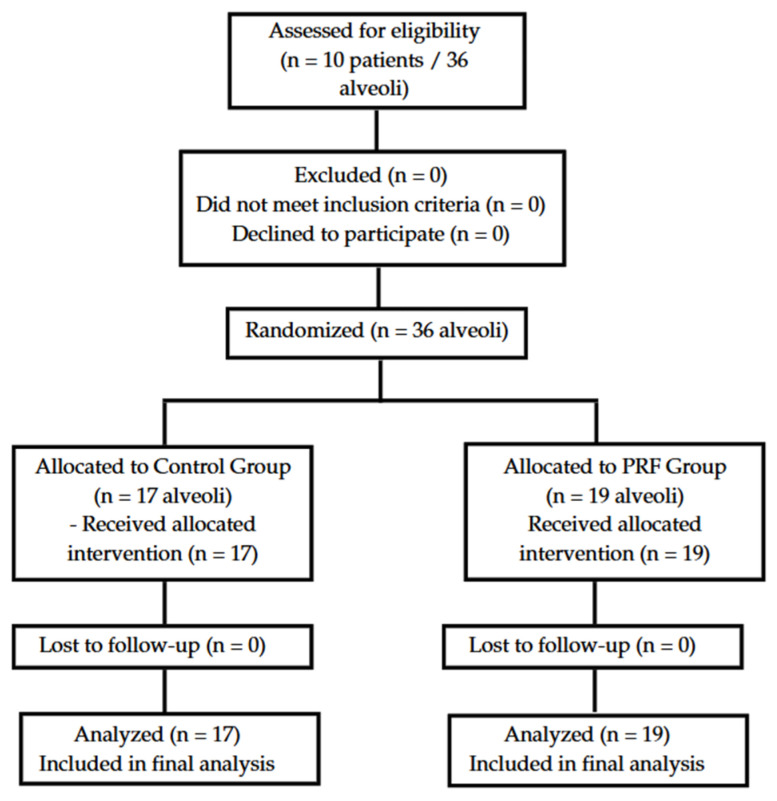
CONSORT flow diagram illustrating patient enrollment, allocation, follow-up, and analysis for the randomized controlled trial. A total of 10 patients were assessed, all met inclusion criteria, and 36 extraction sockets were randomized into two groups: PRF (*n* = 19) and Control (*n* = 17). No losses or exclusions were reported during the follow-up.

Width Measurement Landmarks:

Vestibular and palatal/lingual cortical plates were identified at each reference point (RW-1, RW-3, RW-5) for both baseline and follow-up CBCT images (Figure 2).

Alveolar Height

To determine alveolar height, the following procedure was performed:

A horizontal line (B1) was drawn at the base of the alveolus (bottom cortical limit).

Two additional lines, parallel to the vestibular (BH) and palatal/lingual (LH) cortical plates, were delineated.

The vertical distance between these lines was measured to assess alveolar process height at baseline and follow-up.

Measurements were performed consistently at baseline, 30 days, and 120 days post-extraction.

Bone Tissue Density Assessment

The quality of the newly formed bone tissue was assessed using the gray value scale corresponding to Hounsfield Units (HU), a CBCT-derived measurement method. Although CBCT-based HU values have limitations compared to CT, they provide a descriptive estimation of bone mineralization.

Bone density was evaluated following the protocol adapted from [15]:

Reference Lines

Line B1 was drawn between the apices of adjacent teeth. Line L1 was traced between the cusps of the same adjacent teeth.

Measurement Points

Point A was identified at the bottom of the alveolus.

Point B was located 4 mm apical to B1 along a vertical midline between B1 and L1.

HU values were recorded at 30 and 120 days post-extraction using the same technical acquisition parameters.

All measurements (height, depth at 1, 3, and 5 mm, and bone density in HU) were systematically registered in a standardized Excel 2018 database for statistical analysis.

### 2.6. Outcomes

The outcomes of this randomized controlled trial were assessed through a detailed radiographic analysis using cone beam computed tomography (CBCT), allowing precise three-dimensional evaluation of post-extraction alveolar changes in terms of dimensional stability and descriptive bone density parameters.

Primary Outcome:

The primary outcome was alveolar height preservation, defined as the cephalocaudal distance from the most apical point of the extraction socket to the crestal margin of the alveolar bone. This measurement reflects vertical bone loss or preservation following extraction.

Alveolar height was measured on cross-sectional CBCT views obtained immediately after extraction (baseline), and at 30 and 120 days postoperatively, using standardized anatomical landmarks and consistent imaging parameters (FOV 8 × 8 cm, voxel size 0.2 mm, 120 kVp, 30.89 mAs, exposure time 16 s).

Measurements were performed using Sidexis 4.0 software to ensure reproducibility and accuracy.

Secondary Outcomes:

Two secondary parameters were evaluated:

Alveolar Width:

Vestibulo-palatal (or vestibulo-lingual) width was assessed at three standardized depths: 1 mm, 3 mm, and 5 mm apical to the alveolar crest. Measurements were taken perpendicular to the long axis of the alveolus, from the buccal to the palatal or lingual cortical walls.

These measurements aimed to capture horizontal dimensional changes occurring progressively deeper within the socket, which are relevant for future implant site development.

Bone Tissue Density (Descriptive Analysis):

Bone density within the healing socket was descriptively evaluated through CBCT-derived Hounsfield Unit (HU) values, recognizing the limitations of CBCT for absolute mineral content quantification.

HU measurements were taken at standardized intra-alveolar locations based on anatomical references (e.g., lines B1 and L1), following validated radiographic protocols [15]. Evaluations were conducted at 30 and 120 days post-extraction to monitor radiodensity changes over time, serving as an indirect indicator of bone maturation trends.

No formal statistical comparisons of HU values between groups were performed.

All outcome measurements were conducted by a calibrated, blinded maxillofacial radiologist using the same imaging equipment and software to minimize inter-observer and technical variability.

Statistical Analysis

All statistical analyses were performed using JASP software (version 0.18.1.0), a validated open-source platform designed for robust comparison of clinical datasets in biomedical research.

Descriptive statistics, including means and standard deviations (SD), were calculated for continuous variables to characterize baseline demographics and outcome measures. These descriptive metrics provided an overview of data distribution, central tendency, and variability.

Comparisons between the PRF and control groups at each time point (30 and 120 days) were conducted using independent-samples *t*-tests. This parametric test evaluates whether the means of two independent groups differ significantly, and was applied to outcome variables such as alveolar height, alveolar width at multiple depths, and bone tissue density (Hounsfield Units, HU).

Within-group changes over time (baseline vs. 30 days, and baseline vs. 120 days) were assessed using paired-samples *t*-tests to determine significant differences in each group independently.

To explore the interaction between time and treatment group, a repeated measures analysis of variance (ANOVA RM) was performed. This model assesses whether patterns of change over time differ significantly between groups, incorporating time × group interaction effects to capture the dynamics of regenerative behavior associated with PRF.

Data normality was verified using the Shapiro–Wilk test prior to the application of parametric tests. When assumptions of normality were violated, non-parametric alternatives were considered where appropriate.

The threshold for statistical significance was set at *p* < 0.05. All *p*-values reported are two-tailed.

No subgroup analyses (e.g., stratification by sex or jaw location) or multivariate adjustments were performed, given the small sample size and the homogeneity of the study sample with respect to clinical indication and procedural technique.

## 3. Results

### 3.1. CONSORT Flow Diagram

A total of 10 systemically healthy patients were assessed for eligibility between January and May 2024. All participants met the inclusion criteria and were randomized, resulting in 36 alveoli: 17 allocated to the control group (Group I) and 19 to the PRF group (Group II). All participants received the assigned intervention, completed follow-up, and were included in the final analysis. No losses or exclusions occurred post-randomization. The CONSORT flow diagram illustrating patient progression is shown in Figure 1.

### 3.2. Baseline Characteristics

The initial mean cephalocaudal alveolar height was comparable between groups:

- Group I: 10.45 mm (SD = 1.10)
- Group II: 10.72 mm (SD = 0.84)

There were no statistically significant differences (*t* = −0.853; *p* = 0.399), confirming baseline homogeneity between groups. Complete demographic and clinical characteristics are presented in Table 1.

### 3.3. Primary Outcome–Alveolar Height

At 30 days post-extraction:

- Group II (PRF) showed a significantly higher mean alveolar height (10.61 mm, SD = 0.85) compared to Group I (8.82 mm, SD = 1.68).
- The mean difference was 1.79 mm (*p* < 0.001; 95% CI: −2.674 to −0.901).

**Figure 3:**
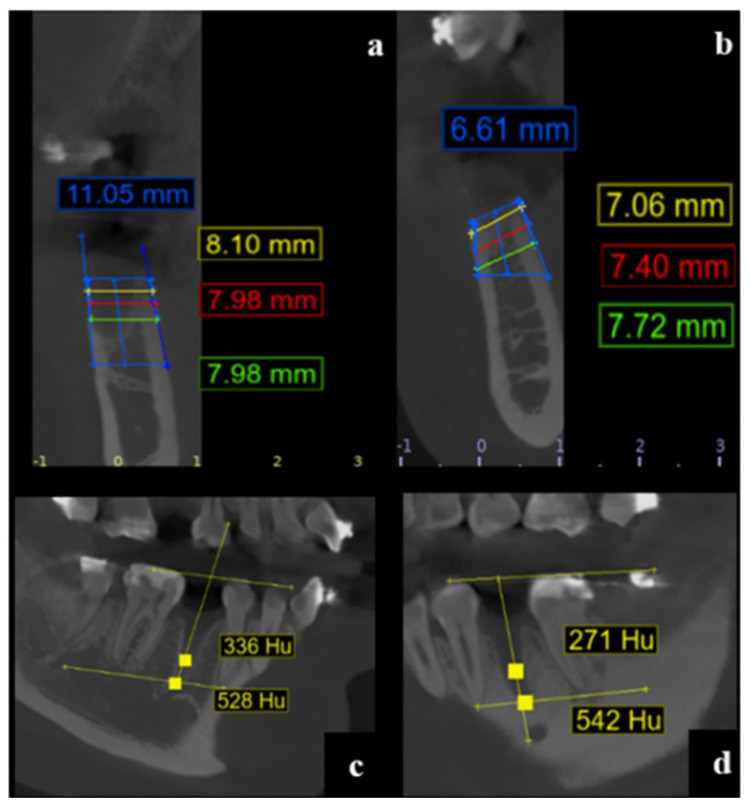
CBCT cross-sectional images at 30 days post-extraction. (**a**) Measurement of alveolar height and socket width at 1, 3, and 5 mm below the alveolar crest in a PRF-treated site (tooth 4.4). (**b**) Corresponding measurements in a control site without PRF application (tooth 3.4). (**c**) Hounsfield Unit (HU) assessment of bone density in the PRF-treated socket. (**d**) Bone density evaluation in the control socket. Measurement points are indicated with white arrows; HU values represent relative mineralization density. Color-coded lines and text indicate the measurement planes and corresponding values: blue for vertical height, red for crestal width, green for basal width, and yellow for in-termediate levels or Hounsfield Unit (HU) values.

These outcomes are illustrated in Figure 3.

**Figure 4:**
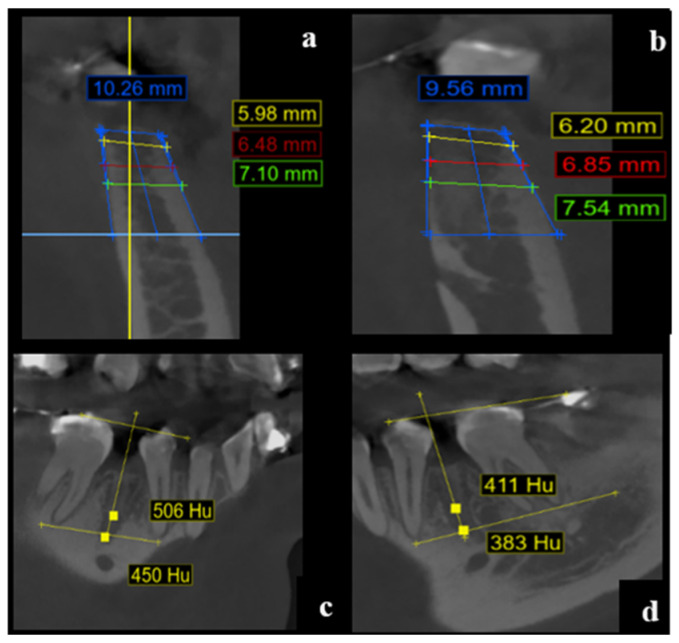
CBCT cross-sectional images at 120 days post-extraction. (**a**) Measurement of alveolar dimensions in a PRF-treated site (tooth 4.4). (**b**) Measurements in a control site (tooth 3.4). (**c**) Bone tissue quality evaluation based on HU values in the PRF group. (**d**) Corresponding assessment in the control group. Images demonstrate vertical and horizontal alveolar changes over time. Color-coded lines and text indicate the measurement planes and corresponding values: blue for vertical height, red for crestal width, green for basal width, and yellow for intermediate levels or Hounsfield Unit (HU) values.

At 120 days post-extraction:

- Group II maintained greater alveolar height (10.30 mm, SD = 1.29) versus Group I (9.31 mm, SD = 1.73).
- However, this difference was not statistically significant (t = −1.197; *p* = 0.059; 95% CI: −2.015 to 0.040). These changes are depicted in Figure 4 and detailed in Table 1.

Intragroup analysis revealed:

- Group I showed a marked decrease in height from baseline to 30 days, with partial recovery at 120 days.
- Group II exhibited minimal variation across time points, indicating superior dimensional stability.

**Figure 5:**
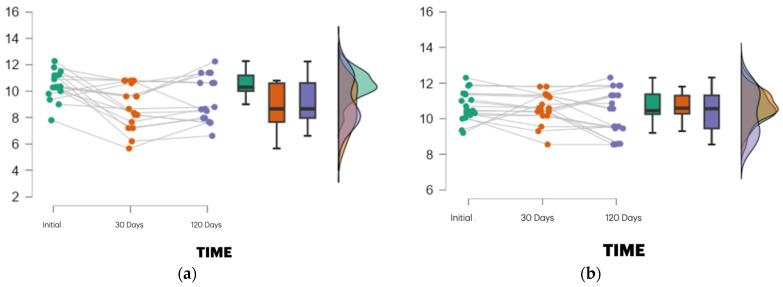
Distribution of alveolar ridge height measurements over time in both study groups. (**a**) PRF group (*n* = 19). (**b**) Control group (*n* = 17). Color code: Green—Baseline (Initial), Orange—30 Days, Purple—120 Days.

These outcomes are illustrated in Figure 5 and detailed in Table 1.

### 3.4. Secondary Outcomes–Alveolar Width and Bone Quality

Alveolar width measurements at 1 mm, 3 mm, and 5 mm depths showed no statistically significant differences between groups at either 30 or 120 days (*p* > 0.05).

At 120 days:

1 mm depth: Group II = 8.06 mm (SD = 1.78); Group I = 8.40 mm (SD = 1.92); *p* = 0.583

3 mm depth: Group II = 8.45 mm (SD = 1.67); Group I = 9.39 mm (SD = 1.69); *p* = 0.101

5 mm depth: Group II = 9.04 mm (SD = 1.65); Group I = 9.99 mm (SD = 1.93); *p* = 0.122

Bone tissue quality, descriptively assessed by Hounsfield Units (HU), ranged from 190 to 282 HU in the PRF group, suggesting denser bone compared to the control group.

However, no formal statistical comparison was performed for HU values.

Full alveolar width and bone quality data are provided in Table 1.

### 3.5. Statistical and Additional Analyses

Repeated measures ANOVA revealed:

▪ A significant time × group interaction for alveolar height (*p* = 0.010; η^2^ = 0.046).
▪ A similarly significant interaction at 1 mm depth (*p* = 0.035; η^2^ = 0.020).
▪ These results are detailed in Table 2

Intergroup analysis indicated:

▪ Group assignment explained 12.5% of the variance in alveolar height (*p* = 0.004), detailed in Table 3.

Post hoc tests showed:

▪ Significant reduction in alveolar height in Group I between baseline and 30 days (−0.630 mm; *p* = 0.005).
▪ Significant reduction in alveolar width at 3 mm (0.800 mm; *p* = 0.027) and 5 mm (*p* = 0.002) depths for Group I.
▪ Group II exhibited a significant decrease in width at 1 mm (−0.968 mm; *p* = 0.004) over time. These results are detailed in Table 4.

### 3.6. Adverse Events

No complications, adverse effects, or unexpected events were reported during the follow-up period. All patients healed uneventfully.

Each panel shows individual patient trajectories (grey lines) along with box plots and vio-lin plots at baseline, 30 days, and 120 days post-extraction. Alveolar height was measured using CBCT based on standardized anatomical landmarks. The PRF group demonstrated a higher median height and reduced variability, particularly at 30 days, compared to the control group, which showed a more pronounced height reduction followed by partial re-covery. The violin plots depict the data density and distribution. These results align with the significant time × group interaction found in the repeated measures ANOVA (*p* = 0.010; η^2^ = 0.046), suggesting enhanced early dimensional stability with PRF. 

## 4. Discussion

This randomized controlled clinical trial aimed to evaluate the impact platelet-rich fibrin (PRF) on alveolar preservation following orthodontic premolar extractions. The findings reinforce the concept that PRF can serve as a biologically active scaffold that promotes early tissue regeneration and bone healing, particularly within the first four to six weeks post-extraction. At 30 days, the PRF group demonstrated a statistically significant improvement in vertical bone height preservation (Δ = 0.92 mm), which aligns with previous reports suggesting that PRF plays a regenerative role by enhancing neovascularization, osteoblastic activity, and early tissue integration [13,14,15,16,19]. This early benefit, attributed to the sustained release of growth factors such The findings reinforce the concept that PRF can serve as a biologically active scaffold that promotes early tissue regeneration and bone healing, particularly within the first four to six weeks post-extraction. At 30 days, the PRF group demonstrated a statistically significant improvement in vertical bone height preservation (Δ = 0.92 mm), which aligns with previous reports suggesting that PRF plays a regenerative role by enhancing neovascularization, osteoblastic activity, and early tissue integration as PDGF, VEGF, and TGF-β, has been corroborated by multiple experimental and clinical studies, highlighting the material’s capacity to modulate both soft and hard tissue healing [5,7,8].

By day 120, although the PRF group continued to show better vertical height maintenance (Δ = 0.84 mm), the difference was no longer statistically significant. This shift suggests that PRF’s regenerative potential may be time-sensitive, with its strongest effects concentrated in the early healing window. This interpretation is supported by Wang et al. [12], who observed that PRF’s influence diminishes over time as the extraction socket progresses into the remodeling phase. Such a pattern implies that while PRF does not prevent long-term bone resorption entirely, it may provide a protective buffer during the critical initial stages when alveolar collapse is most rapid.

Regarding horizontal ridge width, no statistically significant differences were found at 1 mm, 3 mm, or 5 mm depths; however, a consistent, albeit modest, trend favoring the PRF group was noted. These findings resonate with previous literature, including the systematic review by Castro et al. [18] and our own earlier work [20], both of which reported more pronounced effects of PRF in preserving vertical bone dimension and soft tissue contours rather than buccolingual ridge width. This may reflect the biological limitations of PRF in resisting mechanical collapse of thin buccal plates, particularly in sites with pre-existing alveolar fragility or fenestration.

On a biological level, the observed outcomes are mechanistically supported by PRF’s unique structure: a dense fibrin matrix interlaced with platelets, leukocytes, and cytokines that foster cellular migration, angiogenesis, and differentiation. As reported by Ghanaati et al. [21], advanced PRF (A-PRF) and injectable PRF (i-PRF) configurations further enhance this potential by altering the cellular composition and scaffold density, suggesting a modifiable regenerative profile depending on clinical needs. Importantly, PRF differs from traditional bone grafts or membranes in that it relies solely on autologous components, eliminating the risk of immune reactions or foreign body responses while also reducing costs.

CBCT imaging provided additional insight into the regenerative process. Sites treated with PRF exhibited higher mean Hounsfield Unit (HU) values (190–282 HU), potentially reflecting greater bone density and earlier mineralization. Although these radiographic findings were not statistically analyzed due to sample constraints, they align with prior reports by Molina-Barahona et al. [20] and Cardaropoli et al. [22], who identified similar HU increases in PRF-treated sockets. Nevertheless, caution must be exercised when interpreting HU values, as they are influenced by multiple variables, including voxel resolution, scanner calibration, and bone density thresholds [11]. Therefore, while CBCT offers a non-invasive proxy for bone quality, it cannot replace histologic evaluation in confirming true osseous regeneration.

From a clinical perspective, the potential benefits of PRF extend beyond quantitative bone preservation. Studies by Silva et al. [13] and Pradeep [14] have demonstrated that PRF contributes to improved periodontal healing and may even surpass PRP in radiographic bone fill and soft tissue closure. Borie et al. [15] emphasize its versatility in both surgical and non-surgical settings, and Del Corso et al. [16] highlight its application in implantology and maxillofacial bone grafting. Collectively, these findings support the idea that PRF is not merely a filler material, but a bioactive tool that can meaningfully modulate the healing cascade.

Nevertheless, this study is not without limitations. The sample size was relatively small and restricted to young, systemically healthy individuals undergoing planned orthodontic extractions, which limits generalizability to more complex clinical scenarios. Additionally, we did not stratify patients by key anatomical variables such as buccal plate thickness, socket morphology, or baseline bone quality—factors that could influence the effectiveness of PRF. Furthermore, while CBCT was useful in tracking structural changes, histological validation remains essential for accurately assessing true bone regeneration and cellular architecture. Future trials should consider incorporating histomorphometric outcomes, longer follow-up intervals (≥12 months), and diverse patient populations including medically compromised individuals or those with pre-existing alveolar defects.

In summary, PRF emerges from this study as a biologically grounded and user-friendly adjunct for early alveolar preservation. Its ease of preparation, autologous nature, and ability to enhance tissue regeneration without added biomaterials make it an appealing option in both orthodontic and surgical contexts. Although its effects appear to be most impactful during the initial healing period, this early stabilization can have downstream benefits for future implant planning or prosthetic rehabilitation. As regenerative dentistry continues to evolve, PRF holds promise not only as a supportive material but as a key component in personalized, minimally invasive treatment strategies aimed at preserving native bone architecture and optimizing long-term outcomes.

## 5. Conclusions

In conclusion, the findings of this randomized controlled clinical trial suggest that platelet-rich fibrin (PRF) may offer benefits for early alveolar ridge preservation following orthodontic premolar extractions. Specifically:

▪ PRF significantly improved short-term preservation of alveolar vertical height compared to natural healing, particularly within the first 30 days post-extraction.
▪ While PRF-treated sites exhibited a trend toward better horizontal alveolar preservation at 1, 3, and 5 mm depths, no statistically significant differences in width were observed between groups.
▪ CBCT-based evaluation showed qualitatively higher Hounsfield Unit (HU) values in PRF-treated sockets compared to controls; however, as no statistical comparison was performed, these findings should be interpreted cautiously.
▪ The simplicity, autologous origin, and biological safety profile of PRF make it an attractive adjunct in clinical protocols aiming to enhance early socket healing, particularly in orthodontic and implant planning contexts.
▪ Nevertheless, the relatively small sample size and the limitations of CBCT radiodensity assessment must be considered when interpreting the results.
▪ Further multicenter randomized trials with larger, more diverse populations, extended follow-up periods, and histological validation are warranted to consolidate these preliminary findings and to establish evidence-based clinical guidelines.

## Figures and Tables

**Table 1:** Baseline, 30-day, and 120-day measurements of alveolar height and socket width (means ± standard deviations) in PRF-treated and control groups. Between-group comparisons were performed using independent-samples *t*-tests. Statistical significance was established at *p* < 0.05.

Variable	Time	Group	N	M	DE	CV	Averages		t	*p*
							Dif	IC (95%)		
Height	Initial	Group I	17	10.45	1.10	0.11	−0.28	ICI = −0.324 ICS = 0.382	−0.853	0.399
		Group II	19	10.72	0.84	0.08				
	30 days	Group I	17	8.82	1.68	0.19	−1.79	ICI = −2.674 ICS = −0.901	−4.099	<0.001
		Group II	19	10.61	0.85	0.08				
	120 days	Group I	17	9.31	1.73	0.19	−0.99	ICI = −2.015 ICS = 0.040	−1.953	0.059
		Group II	19	10.30	1.29	0.13				
Depth 1 mm	30 days	Group I	17	8.40	1.84	0.22	−0.63	ICI = −1.705 ICS = 0.453	−1.179	0.247
		Group II	19	9.02	1.33	0.15				
	120 days	Group I	17	8.40	1.92	0.23	0.34	ICI = −0.912 ICS = 1.596	0.554	0.583
		Group II	19	8.06	1.78	0.22				
Depth 3 mm	30 days	Group I	17	8.76	1.69	0.19	−0.62	ICI = −1.626 ICS = 0.385	−1.254	0.218
		Group II	19	9.38	1.27	0.14				
	120 days	Group I	17	9.39	1.69	0.18	0.94	ICI = −0.195 ICS = 2.082	1.684	0.101
		Group II	19	8.45	1.67	0.20				
Depth 5 mm	30 days	Group I	17	9.19	1.78	0.19	−0.07	ICI = −1.174 ICS = 1.029	−0.133	0.895
		Group II	19	9.26	1.47	0.16				
	120 days	Group I	17	9.99	1.93	0.19	0.95	ICI = −0.266 ICS = 2.157	1.586	0.122
		Group II	19	9.04	1.65	0.18				

Abbreviations: CV = Coefficient of Variation; Diff = Mean Difference.

**Table 2:** Repeated measures analysis of variance (RM-ANOVA) evaluating intra-group changes in alveolar height and width over time. Effect sizes are reported as eta squared (η^2^). Statistical significance was considered at *p* < 0.05.

Variable	Analysis	Sum of Squares	Degrees of Freedom	Root Mean Square	F	*p*	η^2^
Height	Time	16.45	2	8.224	7.840	<0.001	0.074
	Time ✻ Group	10.26	2	5.132	4.892	0.010	0.046
	Residuals	71.33	68	1.049			
Depth 1 mm	Time	4.21	1	4.212	4.840	0.035	0.020
	Time ✻ Group	4.20	1	4.202	4.828	0.035	0.020
	Residuals	29.59	34	0.870			
Depth 3 mm	Time	0.41	1	0.412	0.627	0.434	0.002
	Time ✻ Group	10.98	1	10.977	16.693	<0.001	0.060
	Residuals	22.36	34	0.658			
Depth 5 mm	Time	1.52	1	1.520	2.284	0.140	0.007
	Time ✻ Group	4.65	1	4.648	6.985	0.012	0.022
	Residuals	22.63	34	0.665			

Abbreviations: F = ANOVA; *p* = Statistical significance; η^2^ = Eta squared. ✻ Time × Group: Interaction term indicating whether the effect of time on the outcome variable differs significantly between treatment groups (PRF vs. control).

**Table 3:** Independent-samples ANOVA results for between-group comparisons at 30 and 120 days. *p*-values and effect sizes (η^2^) are provided to quantify treatment effects.

Variable	Analysis	Sum of Squares	Degrees of Freedom	Root Mean Square	F	*p*	η^2^
Height	Group	27.85	1	27.850	9.800	0.004	0.125
	Residuals	96.62	34	2.842			
Depth 1 mm	Group	0.36	1	0.362	0.071	0.791	0.002
	Residuals	172.50	34	5.073			
Depth 3 mm	Group	0.47	1	0.468	0.107	0.745	0.003
	Residuals	148.11	34	4.356			
Depth 5 mm	Group	3.42	1	3.422	0.663	0.421	0.016
	Residuals	175.46	34	5.161			

**Table 4:** Post hoc pairwise comparisons within each group, assessing changes from baseline to 30 days, and from 30 to 120 days. Results include mean differences, 95% confidence intervals, and corresponding *p*-values. Paired *t*-tests or Wilcoxon signed-rank tests were applied as appropriate.

Group	Variable	Measurement 1	Measurement 2	Dif Root Mean Square	t	*p*	IC Root Mean Square Differences (95%)
Group I	Height	Start	30 days	−1.626	3.692	0.002	ICI = 0.550 ICS = 2.610
		30 days	120 days	0.491	−1.730	0.103	ICI = −1.100 ICS = 0.090
	Depth	30 days (1 mm)	120 days (1 mm)	0.000	0.002	0.998	ICI = −0.755 ICS = 0.755
		30 days (3 mm)	120 days (3 mm)	−0.630	−2.427	0.027	ICI = −1.280 ICS = −0.090
		30 days (5 mm)	120 days (5 mm)	0.800	−3.607	0.002	ICI = −1.355 ICS = −0.465
Group II	Height	Start	30 days	−0.115	0.627	**0.539**	ICI = 0.055 ICS = 0.435
		30 days	120 days	−0.310	1.326	0.202	ICI = −0.300 ICS = 0.970
	Depth	30 days (1 mm)	120 days (1 mm)	−0.968	2.942	0.009	ICI = 0.190 ICS = 1.560
		30 days (3 mm)	120 days (3 mm)	−0.933	3.364	**0.934**	ICI = 0.340 ICS = 1.420
		30 days (5 mm)	120 days (5 mm)	−0.218	0.714	0.218	ICI = −0.480 ICS = 0.900

Abbreviations: Group I = Alveolus without PRF; Group II = Alveolus with PR *p* = Statistical significance; Bold values indicate statistically significant results (*p* < 0.05).

## Data Availability

The data presented in this study are available from the corresponding author upon request.
